# In memoriam: Professor David Cooper (1949 to 2018)

**DOI:** 10.1002/jia2.25152

**Published:** 2018-06-29

**Authors:** Susan Kippax, Kenneth H Mayer, Annette Sohn, Linda‐Gail Bekker, Anton Pozniak, Chris Beyrer, Sharon R Lewin, Owen Ryan, Praphan Phanuphak, Michael Kirby

**Affiliations:** ^1^ Social Policy Research Centre University of New South Wales Sydney Australia; ^2^ The Fenway Institute Fenway Health and Department of Medicine Beth Israel Deaconess Medical Center Harvard Medical School Boston MA USA; ^3^ TREAT Asia/amfAR the Foundation for AIDS Research Bangkok Thailand; ^4^ The Desmond Tutu HIV Centre University of Cape Town Cape Town South Africa; ^5^ Department of Medicine Chelsea and Westminster Hospital NHS Foundation Trust London UK; ^6^ Center for Public Health and Human Rights Johns Hopkins University Baltimore MD USA; ^7^ The Peter Doherty Institute for Infection and Immunity The University of Melbourne and Royal Melbourne Hospital Melbourne VIC Australia; ^8^ Department of Infectious Diseases Alfred Hospital and Monash University Melbourne VIC Australia; ^9^ International AIDS Society Geneva Switzerland; ^10^ HIV‐NAT The Thai Red Cross AIDS Research Centre Bangkok Thailand; ^11^ The Kirby Institute for Infection and Immunity the University of New South Wales Kensington NSW Australia

The recent passing of Professor David Cooper has left the AIDS community bereft of another great soul. David died in Sydney, Australia, on Sunday, 18 March 2018, after a short illness, leaving behind a profound personal and professional legacy.

David's stellar training in Australia and the United States provided him with a strong foundation in clinical research. He was among the first clinical researchers to address the epidemic in Australia in the early 1980s, and became a global leader in developing multinational responses to the challenges of the epidemic. The early cases in Australia, as in many countries, were largely among gay men, then a highly marginalized and stigmatized community. David understood that anti‐gay stigma and discrimination were huge challenges faced by those living with HIV, and he was an early and ardent champion of human rights, inclusion and compassionate care for the community.

In an interview with the Australian Broadcasting Corporation (ABC) in 2015, David reflected that being Jewish perhaps helped him relate to his patients and the stigma they faced. As a 15‐year‐old medical student at the University of Sydney, he never took the value of life for granted. He said: “There were a lot of Jewish kids in the medical school – we were Jews, we were different and we were persecuted because of that. I saw the same sort of things in the vulnerable patients that we dealt with.” 1. Fighting against the stigma and prejudice became a family affair, and David and his then young daughters became Mardi Gras regulars, dressing up as pills fighting the virus.

In 1986, David became the founding Director of the National Centre in HIV Epidemiological and Clinical Research (NCHECR) at the University of New South Wales. The NCHECR was one of the three national research centres established by the Australian government to undertake research that would inform Australia's response to the threat of HIV. Clinical, biomedical, epidemiology and social researchers worked together and with communities affected by HIV to build an accurate picture of HIV transmission and develop strategies to respond effectively to the emerging HIV crisis, in terms of both prevention and treatment. Under his directorship of 32 years, the NCHECR, now named the Kirby Institute in honour of his close friend, The Hon. Michael Kirby, became a world‐class research organization that engages more than 300 researchers.

David's incisive thinking enabled him to lead some of the first studies characterizing primary HIV infection and to develop a clinical trials infrastructure that allowed him and his team and multiple partners across Australia to collaborate on pivotal HIV clinical trials. This included the START trial 2, which provided an important part of the rationale for prompt initiation of treatment upon HIV diagnosis. David used his understanding of the many connections between HIV clinical management and other health conditions when he supported leading investigators in studies of HIV pathogenesis, hepatitis C, management of the cardiovascular and other chronic disease manifestations of HIV, and antiretroviral pre‐exposure prophylaxis.

As the Hon. Michael Kirby noted in a memorial tribute by the University of New South Wales, “David's special gift was having both a huge intellect and a huge heart.” 3. He was a passionate globalist, recognizing the importance of solidarity and mutual support in addressing the global pandemic. He assumed the mantle of international leadership in many ways, serving as the President of the International AIDS Society and on its Governing Council.

At a time when the world's attention was focused on the HIV epidemic in Africa, David recognized the importance of supporting HIV treatment programmes in the Asia‐Pacific. He sought to do that using the methods he relied on throughout his career: prioritizing the needs of people living with HIV, building local capacity, and focusing on science and research. With Joep Lange and Praphan Phanuphak, he had like‐minded colleagues ready to invest in this combination of strategies. Together, they founded the HIV Netherlands Australia Thailand Research Collaboration (HIV‐NAT) in 1996. HIV‐NAT rapidly became a leading centre for innovative HIV clinical trials research in Asia, affirming that the highest standards of HIV basic science, clinical and prevention research could be achieved in a middle‐income country.

A few years later, amfAR was seeking ways to establish its first international HIV research project. When David met with now amfAR CEO Kevin Robert Frost, they shared a vision for a multicentre network that would be the first to collect data on treatment outcomes in people living with HIV in the region. By 2001, the TREAT Asia programme was born. Under the co‐leadership of amfAR and the NCHECR, TREAT Asia grew to include 21 adult and 20 paediatric network sites in 12 countries, working with a range of national, regional and global partner organizations. amfAR has since distributed more than $34 million in grants across the Asia‐Pacific – investments that would not have been possible without David's early mentorship and guidance.

Similar stories could be told about the evolution of Professor Cooper's other research and capacity‐building collaborations, including in Cambodia, Indonesia and Myanmar, the last one launched in July 2017. He was continuously seeking new opportunities to fill knowledge and evidence gaps to improve the quality of care and to support clinicians and scientists. While it is difficult to fully quantify the extent of his individual impact on the regional HIV response, those who worked with him know that the breadth and depth of his contributions made him one of the true giants in our field.

David was also an outstanding mentor, and many investigators and community members frequently sought his wise advice, professional, personal and medical. He trained several generations of basic, clinical and public health researchers and was an outstanding role model as a clinician scientist. His firm belief that research will drive better health outcomes was evident throughout his career.

In addition to being a brilliant scientist, David was a devoted husband and family man. He travelled all over the world with his wife Dorrie, and they enjoyed collecting Clarice Cliff pottery together.

We will remember David as a superlative researcher, but the work was much more to him than gathering data or writing papers – though he continued both activities until his final days. Rather, his primary concern was to make the world a better place, by improving lives, by making an impact on the epidemic. He certainly did that, and more.

## Competing interests

None.

## Authors' contributions

All authors have contributed to the preparation of the manuscript, read and approved the final acknowledgement draft.
